# Optic Neuropathy AFG3L2 Related in a Patient Affected by Congenital Stationary Night Blindness

**DOI:** 10.1155/2024/8581090

**Published:** 2024-11-12

**Authors:** Gabriella Cammarata, Alessandra Mihalich, Emanuela Manfredini, Costanza Lamperti, Stefania Bianchi Marzoli, Anna Maria Di Blasio

**Affiliations:** ^1^Neuro-Ophthalmology Center and Electrophysiology Laboratory, Department of Ophthalmology, IRCCS Istituto Auxologico Italiano, Milan, Italy; ^2^Molecular Biology Laboratory, IRCCS Istituto Auxologico Italiano, Milan, Italy; ^3^Unit of Medical Genetics and Neurogenetic, Fondazione IRCCS Istituto Neurologico C. Besta, Milan, Italy

**Keywords:** case report, CSNB, genetic tests, hereditary optic neuropathy

## Abstract

**Objective:** We describe a patient affected by congenital stationary night blindness (CSNB) secondary to CACNA1F and optic neuropathy associated with an AFG3L2 variant.

**Methods:** We performed comprehensive neuro-ophthalmologic examinations, retinal imaging, complete ocular electrophysiology, and brain and optic nerve MRI. Genomic DNA was extracted from the peripheral blood. The patient's DNA was then investigated by next-generation sequencing (NGS) with a panel including 32 genes associated with retinal dystrophy and therefore with a panel including seven genes associated with genetic forms of optic atrophy.

**Results:** The genetic analysis identified a pathogenetic CACNA1F variant causing CSNB and a heterozygous variant in AFG3L2 that alters OPA1 processing and is known to be associated with OPA1-like optic neuropathy.

**Conclusion:** Optic disc atrophy has been previously described as an atypical feature in the phenotype of CSNB CACNA1F-related. In this patient, we found a variant of the AFG3L2 gene that presumably explains the presence of optic atrophy in a subject affected by CSNB.

**Clinical Relevance:** The clinical evidence of optic atrophy, which is atypical in CSNB, should raise the suspicion of concomitant hereditary optic neuropathy and emphasize the importance of broad genetic diagnostic testing to better define the genotype–phenotype correlation.

## 1. Introduction

Congenital stationary night blindness (CSNB) refers to a genetically determined, largely nonprogressive group of hereditary retinal disorders inherited in an X-linked, autosomal recessive, or autosomal dominant pattern.

The phenotype of CSNB is heterogeneous, with patients more often complaining about dim light vision disturbance or delayed dark adaptation, associated with poor visual acuity, myopia, nystagmus, strabismus, and normal fundus apart from myopic changes [1].

Based on specific full-field electroretinogram (ERG) abnormalities, CSNB can be divided into the complete form (cCSNB or CSNB1) characterized by ON-bipolar pathway dysfunction and the incomplete form (icCSNB or CSNB2) in which there is both ON- and OFF-bipolar pathway dysfunction [1].

The incomplete form of CSNB is associated with pathogenic variants of CACNA1F that encode the *α*-1 SUBUNIT of the voltage-gated L-type calcium channel Ca_v_1.4 and is inherited in an X-linked recessive pattern [2, 3]. The CACNA1F variant causes a reduction in signal transfer from the photoreceptor to the bipolar cells, resulting in a decreased b-wave amplitude in the ERG [1].

*AFG3L2* encodes a protein localized in mitochondria and closely related to paraplegin.

Heterozygous variants in *AFG3L2* are known to cause the autosomal dominant spinocerebellar ataxia 28 (SCA28) [4], whereas, rarely, homozygous *AFG3L2* variants have been associated with a complex autosomal recessive spastic ataxia syndrome (SPAX5) [5].

A previous study demonstrated the pathogenic role of novel heterozygous mutation in AFG3L2 as a relevant cause of both pure and syndromic optic neuropathies in OPA1-negative dominant optic atrophy (DOA) in families or sporadic cases, presenting clinical features nearly indistinguishable from OPA1-related DOA [6], typically characterized by infantile onset and slow progressive bilateral painless central visual loss and pale optic discs usually in the temporal sector as expression of the prevalent papillomacular bundle damage [7, 8].

We describe a patient affected by CSNB secondary to the CACNA1F pathogenetic variant who presented a bilateral optic disc atrophy that was found to be related to another variant in AFG3L2 known as associated with hereditary optic neuropathy. The CACNA1F and the AFG3L2 variants of this patient were previously reported in two different papers [6, 9].

Here, our aim is to underline the clinical relevance of the unusual finding of optic atrophy in the context of CSNB that should raise the suspicion of concomitant hereditary optic neuropathy, emphasizing the importance of broad genetic diagnostic testing.

## 2. Case Description

A 15-year-old boy was evaluated for congenital nystagmus, high myopia corrected since the age of 2, and bilateral low vision. There was no history of vision loss or consanguinity in the family. Vertical nystagmus was noted at 1 month after birth, with progressive improvement starting from 5 years of age and resolution by Age 10. He did not refer to nyctalopia or progressive visual defects.

The patient underwent a full neuro-ophthalmologic examination which included best corrected visual acuity (BCVA), the Ishihara test, Humphrey perimetry (SITA Standard 30-2 program with Size III target, Carl Zeiss Meditec, Dublin, CA), multimodal retinal imaging (Optovue RTvue SDOCT and Heidelberg Spectralis SDOCT), full-field ERG (ERG), pattern ERG (PERG), and visual evoked potential (VEP) obtained with RETIMAX Flash Ganzfeld Color (CSO, Firenze, Italia) according to the International Society for Clinical Electrophysiology of Vision (ISCEV) standards [10–12]. A brain MRI with specific detail on the visual pathway was also later performed to investigate the possible concomitant optic neuropathy responsible for the optic disc pallor.

At the first examination, the cycloplegic refraction was −12.50/−2 (35) RE and −11.00/−1.75 (155) LE, and BCVA was 20/50 OU. Color vision testing was moderately abnormal on the Ishihara test (8/16 RE, 9/16 LE).

**Figure 1:**
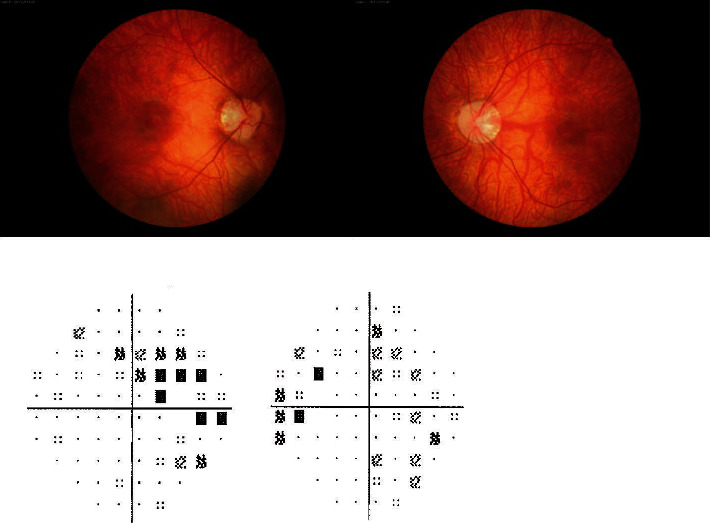
Right and left fundus photographs showing moderate myopic changes, hypochromic retina with abnormal foveal reflex, slight attenuation of retinal vessels, and diffuse pallor of the optic disc; right and left visual field showing bilateral slight generalized relative depression and pericecal scotoma.

Fundus examination revealed moderate myopic changes (tilted disc, mild peripapillary chorioretinal atrophy, myopic peripapillary crescent, diffuse retinal hypopigmentation, and diffuse thinning of the arterial vessel) and marked diffuse pallor of the optic disc. No significant alteration in the fovea or parafoveal area was noted. A slight generalized relative depression and a pericecal scotoma were found on visual field testing in both eyes (Figure 1).

**Figure 2:**
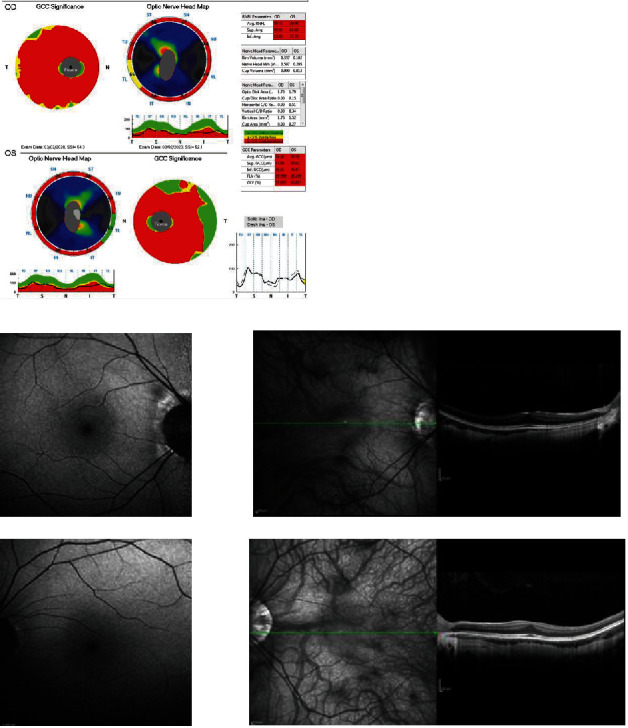
SDOCT showing diffuse thinning of the peripapillary retinal nerve fiber layer and diffusely reduced thickness of the macular ganglion cell complex (Optovue RTvue SDOCT). On fundus autofluorescence (FAF), there is a rarefaction of the physiological hypoautofluorescence, and on structural SD-*OCT* images, the foveal depression is poorly represented with marked thinning of the inner retinal layers and normal thickness and reflectivity of the outer retinal and the retinal pigment epithelium (Heidelberg Spectralis SDOCT).

SDOCT showed diffuse thinning of the peripapillary retinal nerve fiber layer (pRNFL) with an average (Avg microgram) equal to 59.80 in the right eye and 60.77 in the left eye and diffusely reduced thickness of the macular ganglion cells complex (mGCC) with an average value (Avg microgram) of 47.06 in the right and 48.79 in the left eye (Figure 2).

Retinal imaging near-infrared (NIR) showed a hypofluorescent retina with abnormal foveal reflex and slight attenuation of retinal vessels. On fundus autofluorescence (FAF), there was a rarefaction of the physiological hypoautofluorescence, and structural SD-OCT images showed a poorly represented foveal depression with marked thinning of the inner retinal layers and normal thickness and reflectivity of the outer retinal and the retinal pigment epithelium (RPE) layers (Figure 2).

**Figure 3:**
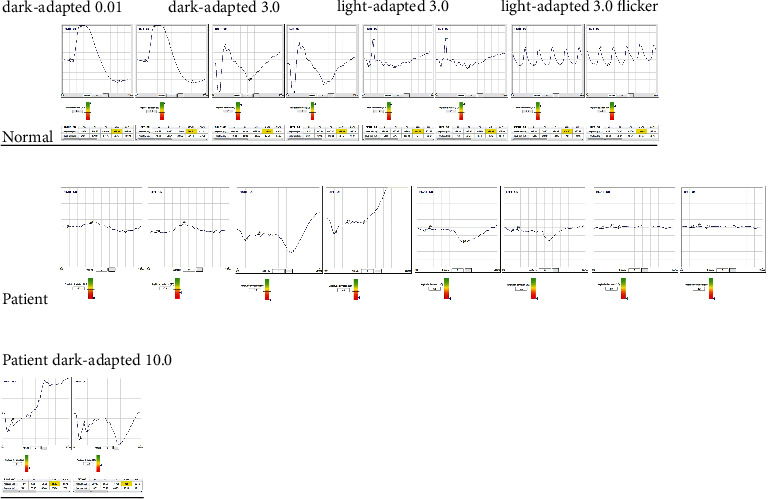
Full-field ERG recorded from the patient compared with normal traces exhibits decreased scotopic and photopic responses with markedly reduced scotopic b-wave amplitudes in the dark-adapted to 3.0 and 10.0, consistent with the negative wave and the typical pattern of incomplete CSNB.

**Figure 4:**
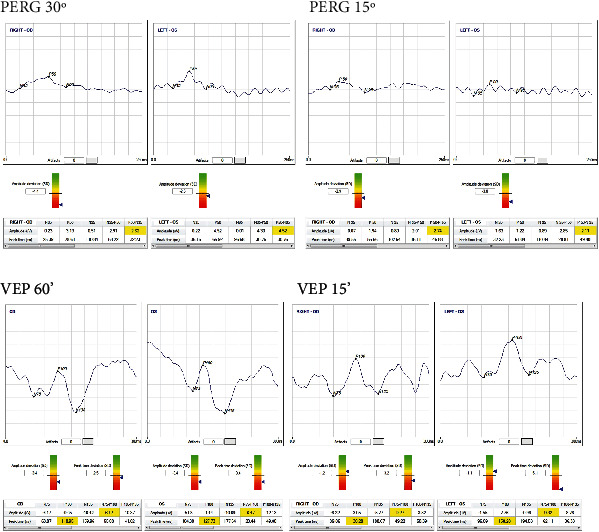
The PERG and the VEP recorded from the patient showing, respectively, bilateral severe macular dysfunction and moderately delayed latencies with reduced amplitude of the cortical responses.

The ERG was notable for decreased scotopic and photopic responses with markedly reduced scotopic b-wave amplitudes in the dark-adapted (DA) 3.0, consistent with an essentially negative wave with the amplitude of the a-wave larger than the b-wave not reaching the baseline [13]. The DA10 was performed later in the follow-up because the time of the first exam predated such an ISCEV indication (Figure 3). The PERG and the VEP showed, respectively, bilateral severe macular dysfunction and moderately delayed latencies with reduced amplitude (Figure 4).

Based on clinical and electrophysiological results, CSNB was suspected, and after the parents of the patients provided informed consent, blood samples were collected from the proband and family members (the parents and the brother). Genomic DNA was extracted and investigated by next-generation sequencing (NGS) with a panel including 31 genes associated with retinal dystrophy (Table S1 in the Supporting Information section), as already reported [9]. The NGS analysis demonstrated the presence in hemizygosis of the duplication in Exon 4 of CACNA1F, ChrX (GRCh37):g.49087408dup NM_005183.4: c.425dupC, resulting in shifting of the reading frame with the insertion of a premature stop codon p.(Val143Glyfs⁣^∗^156). The duplication was located in the S2 Transmembrane Repeat 1 of the protein. The duplication was inherited from the mother, who was an asymptomatic carrier with normal visual function but moderate bilateral reduction of the b-wave on the ERG. The asymptomatic proband's brother does not carry the CACNA1F variant.

**Figure 5:**
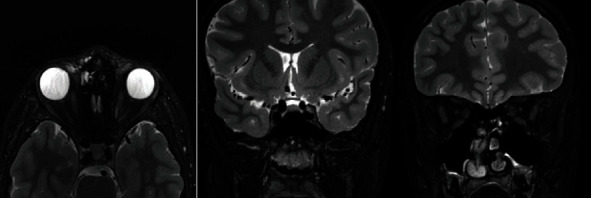
MRI T2 FAT-SAT sagittal and coronal images showing marked thinning of both optic nerves and also even if less of the chiasma and the optic tracts without any signal alteration.

Despite positive testing for the retinal dystrophy, the presence of marked optic disc atrophy not typical for CACNA1F-associated phenotype raised the suspicion of concomitant hereditary optic neuropathy, and the patient underwent MRI with dedicated orbital sequences that showed marked thinning of both optic nerves and, although to a lesser degree, of the chiasma and the optic tracts without signal changes (Figure 5).

The patient was then investigated by NGS with a panel including seven genes associated with genetic forms of optic atrophy: *ACO2*, *AFG3L2*, *CISD2*, *OPA1*, *OPA3*, *RTN4IP1*, and *WFS1* (Table S2 in the Supporting Information section). The genetic analysis demonstrated that the patient harbored a heterozygous missense variant in AFG3L2 Chr18(GRCh37):g.12356821G>A NM_006796.3: c.1036C>T, p.(Leu346Phe) [6], leading to the associated diagnosis of AFG3-related optic neuropathy. This variant is localized in the ATPase domain of the AFG*3L2* clustered in the ATPase domain with other pathogenetic variants causing alteration of OPA1 processing and associated with optic neuropathy, presenting clinical features nearly indistinguishable from OPA1-related DOA [6, 14]. The variant was inherited from the father, who was asymptomatic for visual and neurological disturbances with a normal ophthalmological evaluation except for moderate bilateral cataract, suggesting reduced penetrance. The proband's brother does not carry the AFG3L2 variant.

At 5 years follow-up, the visual function was stable with no significant variation of structural parameters (macular OCT, AF, NIR unchanged; PRNFL Avg *μ*m 56 RE 59 LE; GCC Avg *μ*m 46 RE 56 LE).

The ERG responses were unchanged except for doubt-isolated worsening of the DA 0.01 response in LE and slight deterioration of PERG bilaterally and cortical response (VEP 15⁣′) only in LE, but the functional parameters were not completely reliable due to an excess of artifacts (data not shown).

At this time, we also obtained the DA10 response that confirmed a negative pattern of the ERG (Figure 3).

## 3. Discussion

The patient herewith described presented with atypical clinical features for CSNB due to the finding of bilateral optic nerve atrophy. The atypical phenotype could be caused by the presence of two different variants. The variant in AFG3L2 presumably explains the additional and atypical clinical feature of optic atrophy in the clinical phenotype of CSNB.

Although the phenotype of icCSNB is somehow more heterogeneous than cCSNB, particularly when associated with mutations in CACNA1F, most patients exhibit a nonprogressive clinical course and normal fundi [1]. However, in the past, the presence of progressive disease and optic disc atrophy was already described as very atypical in this clinical setting, but no mutation causative of optic neuropathy was found [15–18].

A novel CACNA1F variant was identified in a young male patient, originally diagnosed with optic atrophy in which the MRI was unremarkable and the presence of mutations in the OPA1 gene was excluded [17].

A retrospective study in 22 males with molecularly confirmed hemizygous mutations in CACNA1F establishes macular inner retinal thinning and optic atrophy as characteristic features of CACNA1F-retinopathy [18].

Another retrospective study in 12 patients with *CACNA1F*-related ocular disease and optic nerve abnormalities supports the hypothesis that CACNA1F could be related to early-onset or congenital optic nerve involvement even without any signs of progressive optic neuropathy [19].

It was then concluded that the mutated CACNA1F gene could itself lead to retinal and optic nerve atrophy with progressive visual loss [17–19].

In this patient, we suggest that the optic atrophy could be the expression of the mitochondrial impairment AFG3L2.

The original study of Caporali et al. demonstrates that the heterozygous variant in *AFG3L2* is a relevant cause of optic atrophy in *OPA1*-negative DOA, impacting about 4% of their cohort [6].

The large majority of these variants affected the AAA (ATPase) domain, suggesting that the unbalanced processing of OPA1 consequent to AFG3L2 dysfunction possibly leads to the optic atrophy phenotype [6, 14].

In our patient, the AFG3L2 variant could explain the optic atrophy as the additional pathologic change associated with the CSNB phenotype, giving new insight into the genotypic–phenotypic correlation.

MRI exhibited a diffuse thinning of the optic pathway (figure), and although brain MRI studies in hereditary retinal dystrophy have shown structural changes within the visual pathways [20] that have been interpreted as secondary, these changes could also indicate a primary manifestation of an associated neurodegenerative optic nerve disease.

The absence of a progressive clinical course in the 5-year follow-up could be attributed to the short observation interval.

It obviously remains difficult to draw firm conclusions from a single case, and it will be necessary to observe the evolution over time to better define the genotype–phenotype correlation, but the optic nerve involvement in *CACNA1F*-related disease needs a more accurate diagnostic for a gene-based deep-phenotyping approach and for the development of novel therapy.

## 4. Conclusion

The finding of the AFG3L2 variant could explain the presence of optic atrophy as an associated atypical feature for CSNB and emphasizes the importance of broad genetic diagnostic testing, including investigation of potential new variants in another gene or modifier alleles that may contribute to determining the uncommon feature of hereditary retinal diseases.

Additional cases must be gathered to understand the genotype–phenotype correlation in CACNA1F associated with optic atrophy.

## Data Availability

The data that support the findings of this study are available from the corresponding author upon reasonable request.

## Supporting Information

Supporting Information Additional supporting information can be found online in the Supporting Information section. Table S1. Genetic screening for retinal dystrophy. Table S2. Genetic screening for optic neuropathy.
