# Supplementary material for: Optic Neuropathy AFG3L2 Related in a Patient Affected by Congenital Stationary Night Blindness
Source: Case Rep Ophthalmol Med. 2024 Nov 12;2024:8581090. doi: 10.1155/2024/8581090 (PMC11576081; doi:10.1155/2024/8581090)
Supplement: Supporting Information — Additional supporting information can be found online in the Supporting Information section. Table S1. Genetic screening for retinal dystrophy. Table S2. Genetic screening for optic neuropathy. [file 8581090.f1.docx]

**Genetic Screening for Retinal dystrophy (Tab S1)**

| Gene | cDNA sequence | RefSeqGene |
| --- | --- | --- |
| *ABCA4* | NM_000350.2 | NG_009073.1 |
| *ADGRV1* | NM_032119.3 | NG_007083.2 |
| *BEST1* | NM_004183.3 | NG_009033.1 |
| *CACNA1F* | NM_005183.3 | NG_009095.2 |
| *CDH23* | NM_022124.5 | NG_008835.1 |
| *CHM* | NM_000390.3 | NG_009874.2 |
| *CNGA3* | NM_001298.2 | NG_009097.1 |
| *CNGB3* | NM_019098.4 | NG_016980.1 |
| *CRX* | NM_000554.5 | NG_008605.1 |
| *ELOVL4* | NM_022726.3 | NG_009108.1 |
| *EYS* | NM_001142800.1 | NG_023443.2 |
| *GNAT2* | NM_005272.3 | NG_009099.1 |
| *GUCA1A* | NM_000409.4 | NG_009938.1 |
| *GUCY2D* | NM_000180.3 | NG_009092.1 |
| *IMPDH1* | NM_000883.3 | NG_009194.1 |
| *MYO7A* | NM_000260.3 | NG_009086.1 |
| *NR2E3* | NM_014249.3 | NG_009113.2 |
| *PCDH15* | NM_033056.3 | NG_009191.2 |
| *PRPF3* | NM_004698.3 | NG_008245.1 |
| *PRPF31* | NM_015629.3 | NG_009759.1 |
| *PRPH2* | NM_000322.4 | NG_009176.1 |
| *RHO* | NM_000539.3 | NG_009115.1 |
| *RLBP1* | NM_000326.4 | NG_008116.1 |
| *RP1* | NM_006269.1 | NG_009840.1 |
| *RP2* | NM_006915.2 | NG_009107.1 |
| *RPE65* | NM_000329.2 | NG_008472.1 |
| *RPGR* | NM_000328.2 | NG_009553.1 |
| *RS1* | NM_000330.3 | NG_008659.3 |
| *USH1C* | NM_005709.3 | NG_011883.1 |
| *USH1G* | NM_173477.4 | NG_007882.2 |
| *USH2A* | NM_206933.2 | NG_009497.1 |

**Genetic Screening for optic neuropathy (Tab S2)**

|  | \|  \|  \| \|  \| \| \| --- \| --- \| --- \| --- \| --- \| \| Gene \| \| cDNA sequence \| \| RefSeqGene \| \| \| *ACO2* \| \| NM_001098.3 \| \| NG_032143.1 \| \| \| *AFG3L2* \| \| NM_006796.3 \| \| NG_023361.1 \| \| \| *CISD2* \| \| NM_001008388.5 \| \| NG_008636.2 \| \| \| *OPA1* \| \| NM_015560.3 \| \| NG_011605.1 \| \| \| *OPA3* \| \| NM_025136.4 \| \| NG_013332.1 \| \| \| *RTN4IP1* \| \| NM_032730.5 \| \| NG_047205.1 \| \| \| *WFS1* \| \| NM_006005.3 \| \| NG_011700.1 \| \| \|  \|  \| \|  \| \| \|  \|  \| \|  \| \| \|  \|  \| \|  \| \| \|  \|  \| \|  \| \| \|  \|  \| \|  \| \| \|  \|  \| \|  \| \| \|  \|  \| \|  \| \| |  |
| --- | --- | --- | --- | --- | --- | --- | --- | --- | --- | --- | --- | --- | --- | --- | --- | --- | --- | --- | --- | --- | --- | --- | --- | --- | --- | --- | --- | --- | --- | --- | --- | --- | --- | --- | --- | --- | --- | --- | --- | --- | --- | --- | --- | --- | --- | --- | --- | --- | --- | --- | --- | --- | --- | --- | --- | --- | --- | --- | --- | --- | --- | --- | --- | --- | --- | --- | --- | --- | --- | --- | --- | --- | --- | --- | --- | --- | --- | --- | --- | --- | --- | --- | --- | --- | --- | --- | --- | --- | --- | --- |
|  |  |  |
|  |  |  |
|  |  |  |
|  |  |  |
|  |  |  |
|  |  |  |
|  |  |  |
